# The Effect of Weak Confinement on the Orientation of Nanorods under Shear Flows

**DOI:** 10.3390/nano8030130

**Published:** 2018-02-26

**Authors:** Saman Monjezi, James D. Jones, Alyssa K. Nelson, Joontaek Park

**Affiliations:** The Department of Chemical & Biochemical Engineering, Missouri University of Science & Technology, Rolla, MO 65409, USA; saman.monjezi@mst.edu (S.M.); jdjzq4@mst.edu (J.D.J.); akn5y3@mst.edu (A.K.N.)

**Keywords:** rod-like particle, nanorod, orientation distribution, orientation moments, microchannel flow

## Abstract

We performed a numerical analysis to study the orientation distribution of a dilute suspension of thin, rigid, rod-like nanoparticles under shearing flow near a solid boundary of weak confinement. Brownian dynamics simulation of a rod was performed under various ratios of shear rate and rod diffusivity (Peclet number), as well as the center-of-mass position (wall confinement). We discuss the effects of Peclet number and wall confinement on the angle distributions, Jeffery orbit distribution and average orientation moments. The average orientation moments, obtained as a function of Peclet number and wall confinement, can be used to improve a previous shear-induced migration model. We demonstrate that the improved model can give excellent prediction of the orientation moment distributions in a microchannel flow.

## 1. Introduction

There have been multiple studies performed on the orientation dynamics and distributions of rod-like micro/nanoparticles in shear flow because these affect the center-of-mass distributions and rheological properties of the suspension of rod-like particles [1,2]. With rapidly advancing applications of micro/nanoparticles, which have shape-specific properties, it is becoming increasingly important to understand the structure and dynamics of micro or nano-sized rod-like particles or macromolecules in microscale flow systems [3,4,5,6,7,8]. However, in contrast to the various studies on the orientation and distribution of rod-like particles, theories on the distribution of rod-like particles near solid boundaries of a microscale flow are not enough to clarify abnormal experimental behaviors. For example, the elution order of gold nanorods in field-flow fractionation, which is a particle separation technique [9], is not clearly understood yet [10,11]. Therefore, a more accurate calculation of rod distribution under consideration of the steric effect of a wall is required for the prediction of the dynamics and elution behaviors in such a system [12,13]. In this work, we focus on the steric effect of a wall on the rod orientation distribution, more specifically confined in a channel, of which height is larger than the long axis length of a rod. 

Rotation of a non-Brownian rod in an unbounded shear flow was found to follow a trajectory called Jeffery orbit [14]. Several works have shown that the Jeffery orbit is affected by hydrodynamic and mechanical interactions with other rods, Brownian motion and inertia [15,16,17,18,19]. For Brownian rods in a shear flow, Boeder [20] suggests an equation to describe the orientation distribution of a rod. That distribution can be characterized by the ratio between the shear rate, $\dot{\gamma }$ and the rotational diffusivity of the rod, *D_R_*, which is defined as Peclet number:

(1) $$Pe\equiv \frac{\dot{\gamma }}{D_{R}}$$

The orientation distribution can be numerically solved as a function of *Pe* [21]. The average values of orientation moments (the products of the orientation vector components) of a Brownian spheroid as a function of *Pe* were calculated, as well as derived in a form of harmonic potential [22,23]. It was also shown that the average orientation moments obtained by performing Brownian rod simulation of a slender body are very similar to those of a spheroid [24]. The average orientation moments were also used in a model equation for predicting the center-of-mass distribution influenced by shear-induced migration [25,26,27]. However, the average orientation moments when considering the effect of the wall were not available, which resulted in a discrepancy between the simulated and the analytically derived distributions [25,26], also shown in Figure 16.

The steric hindrance effect on the rod orientation was studied for a strongly confined channel with very narrow height (less than the long axis length of a rod) [28]. However, the study for a weakly confined channel with a wide height (larger than the long axis length of a rod) gives only limited information which is not enough to be applied to the aforementioned predictions of rod behaviors [29]. Moreover, these studies were performed on an assumption that rod rotation is on a 2D plane, excluding the vorticity direction. There were also studies performed on rod orientation and its effect on the distributions in limited flow conditions, such as low *Pe* [30,31,32]. 

The lack of study on this issue is likely because the effect is not easily characterized by the distance between a rod’s center-of-mass and the wall surface, *r*_c_, due to the combined translational and rotational motion as a response to a collision with the wall. For example, once the tip of a rod touches the wall, two types of behavior are possible: either its *r*_c_ changes, or its *r*_c_ remains the same with a change in its orientation. Hijazi and Khater studied both cases (named “surface restitution”) via Brownian dynamics simulation and suggested that the response having a change in *r*_c_ is the more reasonable of the two outcomes [28,29]. Additionally, it has been known that a rod under a shear flow near a wall shows “pole-vault” type rotation, which accompanies the lift of *r*_c_ due to the excluded volume effect of the wall [33,34,35]. 

Our study will systematically show the orientation distributions in terms of normalized probability distribution functions of various angles in wide ranges of *Pe*. The details of our simulation algorithm will be described in the next section. The simulation results will be presented in terms of various orientation distributions and the average orientation moments as a function of *Pe* with various confinements (i.e., given values of *r*_c_). Finally, it will be demonstrated that our study can be applied to show an improved prediction of the average orientation of a Brownian rod flowing in a microchannel than the previous works [24,25,26].

## 2. Numerical Method 

### 2.1. Definitions of the Variables for a Rod Configuration

**Figure 1:**
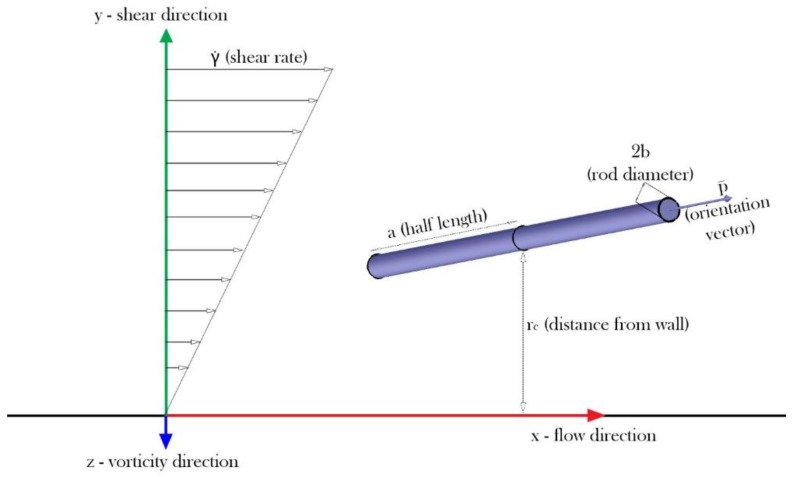
Schematic diagram of a rod under shear flow near a wall.

For the investigation of a rod orientation restricted by a wall, we performed Brownian dynamics simulation of a thin, neutrally buoyant, rigid rod near a wall in a simple shear flow. As shown in Figure 1, a rod with its principal axis length, *L* = 2*a* and its diameter, *d* = 2*b*, is under a flow with a shear rate of $\dot{\gamma }$. The Cartesian coordinate system is set so that the flow is in the *x*-direction, the velocity gradient is in the *y*-direction and the vorticity is in the *z*-direction. It is assumed that the channel height, *H*, is larger than 2*L* so that the rod orientation is only restricted by the bottom wall (*y* = 0). The channel width is much larger than the channel height so that the steric effect in the *z*-direction is ignored. The unit vector describing rod orientation is **p** and has *p*_x_, *p*_y_ and *p*_z_ components in the respective *x*, *y* and *z* directions. The rod configuration is approximated as a slender-body [36] and thus its rotational diffusivity can be written as follows:

(2) $$D_{R}=\frac{3k_{B}T}{8\pi \mu a^{3}}\ln\left(\frac{2a}{b}\right)$$

Here, *k_B_* is the Boltzman constant, *T* is the absolute temperature and *μ* is the solvent viscosity. 

**Figure 2:**
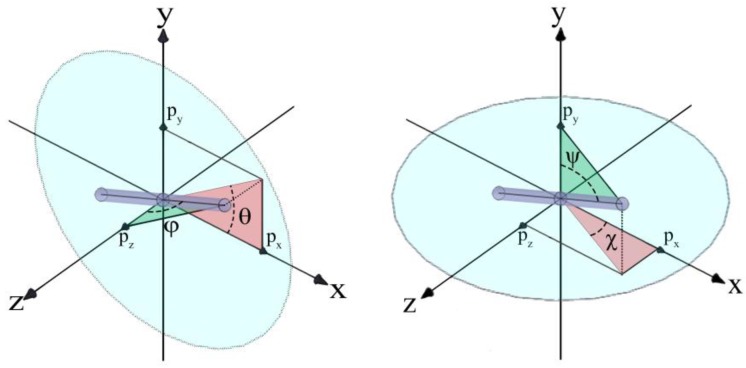
The orientation variables for a rod configuration. (**Left**) *θ* and *ϕ* as well as (**Right**) *ψ* and *χ*. Note that either set of *θ* and *ϕ* or *ψ* and *χ* determines the rod orientation **p**. The distribution of *θ* gives a unique feature (asymmetric distribution) of Brownian rod under shear flow. The distribution of *ψ* is directly related to the geometrical constraint by the weak confinement.

Figure 2 demonstrates the angles that were investigated: *θ* is the angle between a rod’s principal axis and the flow direction on the *xy*-plane and *ψ* is the angle between a rod’s principal axis and the shear direction (*y*). We focus on the distributions of *θ* and *ψ* which show characteristic rod orientation behaviors. However, we also define the other angles: *ϕ* is the angle between a rod’s principal axis and the vorticity direction (*z*), *χ* is the angle between a rod’s principal axis and the flow direction on the *xz*-plane. Note here that *χ* is not affected by the confinement. The relations between these angles and the vector components of **p** can be written as shown below:

(3) $$\theta =\tan^{-1}\left(\frac{p_{y}}{p_{x}}\right),\;\;\;\varphi =\cos^{-1}\left(p_{z}\right),\;\;\chi =\tan^{-1}\left(\frac{p_{z}}{p_{x}}\right),\;\;\mathrm{and}\;\psi =\cos^{-1}\left(p_{y}\right)$$

### 2.2. Simulation Approach and Assumptions

**Figure 3:**
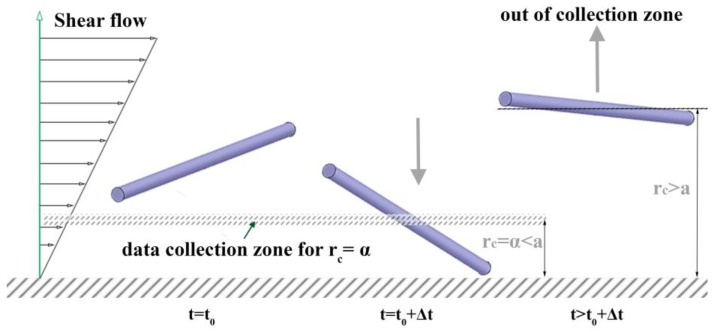
Schematic demonstration of a rod movement in a microchannel near a wall and the rod orientation data collection algorithm in the previous simulation by Park & Butler (2009).

For a Brownian rod experiencing a weakly confined channel flow, its *r*_c_ continues to change dynamically due to Brownian translational motion and collisions with the wall (see Figure 3). Therefore, the proper algorithm must be implemented to correctly characterize the wall confinement effect on the rod orientation in terms of *r*_c_ = *α*, the given position of interest. 

Theories and simulation approaches for Brownian dynamics of rods have been developed by many researchers [37,38]. Park & Butler (2009) performed a simulation of a Brownian rod in a microchannel shear flow while considering long-range as well as short-range (lubrication) hydrodynamic interactions between a rod and the walls. The main purpose of the simulation was to confirm the center-of-mass distribution in the cross-sectional direction predicted by a previous analytical model. The orientation distribution in the cross-sectional direction was also investigated using the simulation data. Comparing the simulation results that both considered and ignored hydrodynamic interactions, it was found that the average orientation moments did not show any noticeable differences, even in the near-wall region. It was conjectured that the excluded volume effect on particle distribution is more dominant than the hydrodynamic interaction in the near-wall region. This result suggests that although the hydrodynamic interaction affects each rod’s motion the resulting averaged orientation distribution is not affected. Moreover, our interest is more focused on the steric effect on the orientation distribution and moments. Therefore, hydrodynamic interaction is not considered in our simulation method.

**Figure 4:**
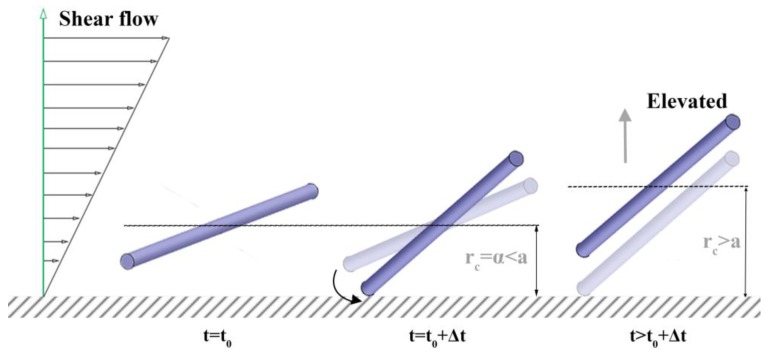
Schematic description for the “Brownian collision” event: Once a tip of a rod invades a boundary, the *r*_c_ of the rod is lifted without changing its **p**.

**Figure 5:**
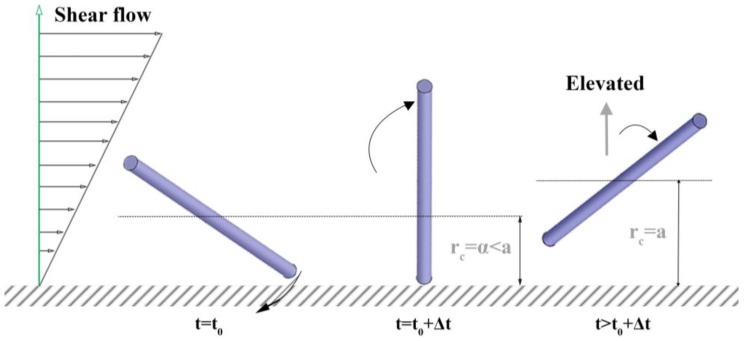
Schematic description for the “shear collision” event and the subsequent “pole-vault motion”. This motion suddenly pushes *r*_c_ from *α* to *a*.

A rod in the near-wall region (0 < *r*_c_ < *a*) can collide with a wall due to either Brownian motion or shear flow. Hijazi and Khater [28,29] classified the types of rod collisions with a wall as Brownian collision and shear collision in their “surface restitution” study. They also investigated how the rod translation and rotation are changed by the collisions. They showed that it is plausible for the Brownian collision, either caused by Brownian translation or rotation, to result in a rod translation away from a wall (lift of *r*_c_), as shown in Figure 4, considering a theoretical center-of-mass distribution. They also claimed that their experiment observed the shear collision to result in the pole-vault type, as also observed by others [33,34,35], rotation which lifts *r*_c_ to *a*, as shown in Figure 5. Either collision results in the lift of *r*_c_: the orientation after the lift is no longer equal to the orientation at the original rod position of interest, *r*_c_ = *α*. Furthermore, the lifted rod comes back to the original position *r*_c_ = *α* by Brownian translation later in the simulation, which is expected to make the orientation at collision and at returning more unrelated. 

**Figure 6:**
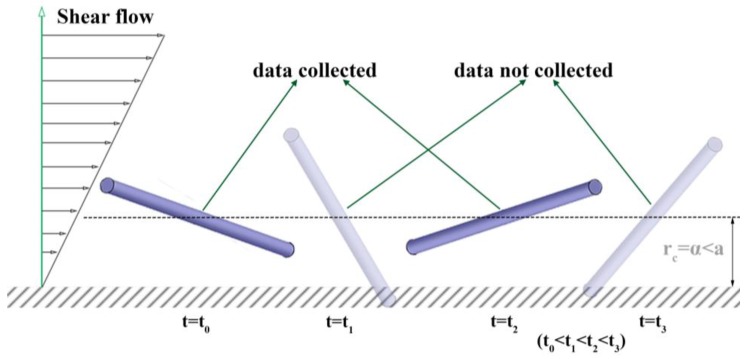
Schematic demonstration for the data collection algorithm in the simulation method proposed in this study.

Based on those two arguments, considering the relative frame on a rod, we propose to study the steric effect of a wall on the rod orientation distribution by investigating the rod orientation data collected through the simulation of Brownian rotation of a rigid rod of which *r*_c_ is fixed at a chosen position, *r*_c_ = *α*. During the simulation, if the tip of a rod invades the boundary (|*p*_y_| > *α*/*a*), the resulting configuration data will not be collected for analysis (shown in Figure 6). Our assumption is that the orientation data collected in the previous simulation method (Figure 3) and our proposed method (Figure 6) are equivalent or at least acceptably close. We chose the proposed method to investigate the effect of the distance from a wall, *α*, on the orientation distribution and average moments more systematically and efficiently. The previous simulation had a difficulty in collecting enough number of data because the probability for a rod existence (the center-of-mass distribution) in the near-wall region is lower due to the shear-induced migration. The resulting orientation distributions from this simulation and the previous simulation will be compared with each other to confirm the validation of the assumption stated above, which will be shown in the Results & Discussion section. It is also important to mention that we tried multiple different simulation methods. For example, we applied excluded volume force or re-assign a random orientation after a collision. Although those methods seem intuitively reasonable, they all gave unphysical results, which imply the validation of our proposed method. 

### 2.3. Initial Configuration

For each simulation *r*_c_ = *α* is chosen to be between 0 and *a* and *Pe* is chosen to be between 10^−3^ and 10^4^. Furthermore, an initial orientation of a rod is randomly determined through the following stepwise procedure [39]:

(1) *p*_x_, *p*_y_ and *p*_z_ are assigned a random number between −1 and 1.
(2) If |**p**| > 1, repeat step (1). Otherwise, normalize *p*_x_, *p*_y_ and *p*_z_ with |**p**|.
(3) If the normalized *p*_y_ is not between −*α*/*a* and +*α*/*a*, repeat steps (1) and (2) until *p*_y_ is correctly constrained (−*α*/*a* ≤ *p*_y_ ≤ +*α*/*a*).

### 2.4. Equation of Motion

The rotation of a Brownian rod under a shear flow can be described by the following equation:

(4) $$\dot{p}=\dot{\gamma }p_{y}\left(\hat{x}-p_{y}p\right)+\frac{3}{\pi \mu L^{3}}\ln\left(\frac{2L}{d}\right)\left[T\times p\right]$$

Here, $\hat{x}$ is a unit vector in the *x*-direction. Brownian torque is denoted as T. With some manipulation, as described in the previous work by Park [26], a new orientation can be calculated numerically at each time step by integrating the following equation.

(5) $$\dot{p}=p_{y}\left(\hat{x}-p_{y}p\right)+\sqrt{\frac{2}{Pe\Delta t}}\left(I-pp\right)\cdot w-\frac{2p}{Pe}$$

Here, *t* is a dimensionless time in terms of a characteristic time of 1/$\dot{\gamma }$. The identity matrix is ***I***. A random vector, ***w***, has a mean of zero and one unit variance [39]. The third term on the right hand side is a correction term for numerical integration by a modified Euler method, which reduces computational time because it does not require correction at the intermediate time step [40]. 

### 2.5. Sampling Data during Dynamic Simulation

The integration of Equation (5) is repeated from *t* = 0 to *t*_end_, the end time for one particle simulation. It is then repeated for *N* particles. During that “one simulation set” over *N* particles for each period of *t*_end_, a rod configuration is sampled in terms of **p** at each *m*-th sampling time for the *n*-th particle, *t*_n,m_,. If the sampled |*p*_y_(*t*_n,m_)| is less than *α/a* (i.e., the rod configuration is within the confinement), the orientation data is collected for analysis (see Figure 6). We confirmed that the effects of the chosen simulation parameters give convergent results. It is also important to note here that the invasion of the wall boundary is evaluated based on the rod center line, neglecting the rod diameter. Details of a rod geometry (such as cylinder or spheroid) may be only important for low values of *a*/*b* < 10. For thin slender rods, *a*/*b* > 10, the diameter can be neglected or adjusted easily, which will be shown in the application to modification of a shear-induced migration model. 

### 2.6. Orientation Distribution

Rod orientation distributions were investigated by plotting the rod angles from the collected orientation data determined from the Brownian dynamics simulation. The collected rod configuration data, **p**(*t*_n,m_), was converted for each angle via Equation (3) to obtain probability distribution functions (PDFs). The converted angle data, *θ*(*t*_n,m_) and *ϕ*(*t*_n,m_), are counted on each unit area (∆*θ* = 1° by ∆*ϕ* = 1°) on a spherical surface spanned by the tips of a rod. The counted bins on each unit area are then normalized by the total number of the collected sampled data to give the PDF on the spherical surface. In other words, integration of the PDF on the whole range gives 1. Additionally, each angle is counted on unit interval (∆angle = 1°) and then normalized to give the PDF of the corresponding angle. The simulation parameters were chosen as ∆*t* = 5 × 10^−7^, *t*_end_ = 100 and *N* = 1000. The sampling was made at each time step. 

### 2.7. Average Orientation Moments Calculation

Orientation moments were averaged over the collected orientation data, **p**(*t*_n,m_). For example, an ensemble average of one of the second-order orientation moments, <*p*_x_*p*_y_>, obtained from the one simulation set is:

(6) $$\langle p_{x}p_{y}\rangle =\frac{1}{N}\sum_{n}^{N}\frac{1}{M\left(n\right)}\sum_{m}^{M\left(n\right)}p_{x}\left(t_{n,m}\right)p_{y}\left(t_{n,m}\right)$$

Here, *M*(*n*) is the total number of the collected orientation data sets falling within the boundary for the *n*-th particle simulation. The average values from Equation (6) typically have large standard deviations for low *Pe*’s due to the broad orientation distribution. Because we intend to extract a model for each of the average moments in terms of *Pe* and *α*, a different approach was adapted to get more convergent values with smaller standard deviations. We used ∆*t* = 5 × 10^−7^, *t*_end_ = 1000 and *N* = 100. Data was sampled at every 200th time step. The determination of this “one simulation set” was repeated until five ensemble average values were obtained using Equation (6). These five values were then averaged again. Most of the resulting standard deviations determined from this method were less than 2% of the average values. 

We calculated all of the possible combinations of the second-order and the fourth-order orientation moments. However, we only display <*p*_x_*p*_y_>, <*p*_y_^2^> and <*p*_x_*p*_y_^3^>, which are related to a theoretical model equation for shear-induced rod migration [12,13,25,26].

## 3. Results and Discussion

### 3.1. Orientation Distribution near a Wall

PDFs of *θ*, *ϕ*, *ψ* and *χ* were obtained from each simulation, as well as PDFs of the spherical surface spanned by the tips of a rod for various values of *Pe* and *α*.

**Figure 7:**
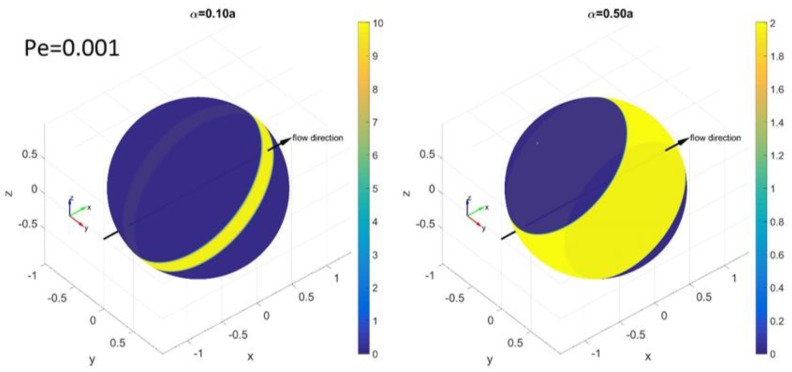

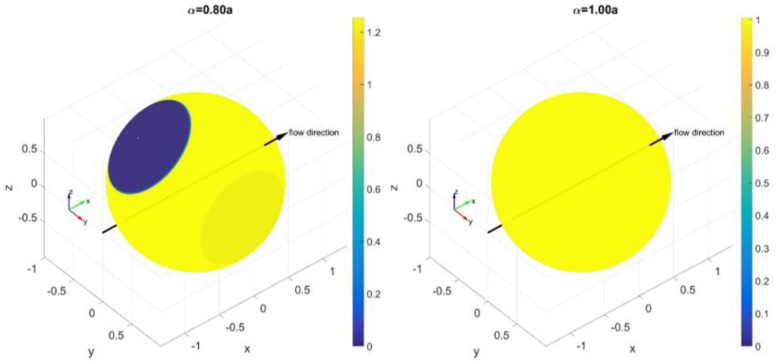
Simulation results for the PDF(*θ*,*ϕ*) on the spherical surface of the tips of a rod at *Pe* = 0.001 with *α*/*a* = 0.1, 0.5, 0.9 and 1.0. The color bars represent the probability density levels of each PDF from yellow (highest probability) to dark blue (lowest probability) (color online).

**Figure 8:**
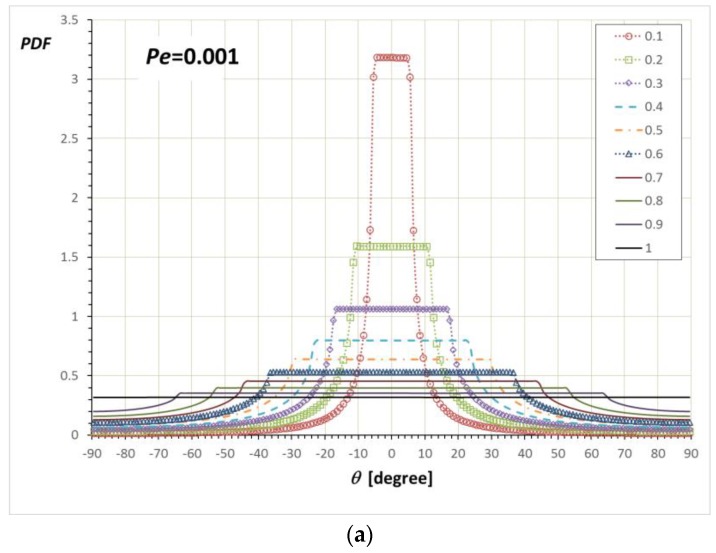

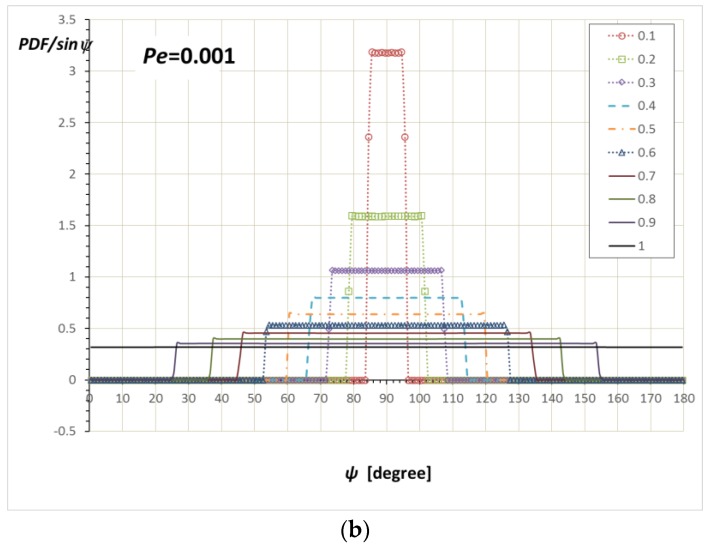
Simulation results for the (**a**) PDF(*θ*) and (**b**) PDF(*ψ*)/sin*ψ* at *Pe* = 0.001 with various *α*/*a*.

Figure 7 and Figure 8 show PDFs at *Pe* = 0.001. At this very low value of *Pe* the effect of shear on each PDF is negligible and the effect of Brownian rotation dominates the PDF. Figure 7 shows the spherical PDF(*θ*,*ϕ*) at *Pe* = 0.001. If there is no confinement, (*α*/*a* ≥ 1), the PDF becomes almost evenly distributed over the spherical surface due to Brownian rotation. As the confinement is varied with *α*/*a* = 0.1, 0.5, 0.9 and 1.0, the PDF gets restricted within the confinement but the restricted distribution is still even. 

Figure 8 shows PDFs for *θ* and *ψ* defined in Equation (3). Figure 8a shows the PDF(*θ*) at *Pe* = 0.001. For the unbounded case of *α*/*a* = 1.0, the PDF(*θ*) is also almost evenly distributed. As *α*/*a* decreases, the values of PDF between confinement angles, $\sin^{-1}\left(-\alpha /a\right)<\theta <\sin^{-1}\left(+\alpha /a\right)$, increase in height but is still almost evenly distributed. Less probable distribution outside of the confined angle region is possible for the configurations near the *z*-axis. For example, although **p** = (0, 0.5, 0.866) has *θ* = 90°, this orientation can exist out of any *θ* confinement region. The PDF(*ψ*) is only non-zero inside of the confinement angle range, $\cos^{-1}\left(+\alpha /a\right)<\psi <\cos^{-1}\left(-\alpha /a\right)$. Therefore, the PDF(*ψ*) at each confinement looks similar to squares within that confinement range. 

**Figure 9:**
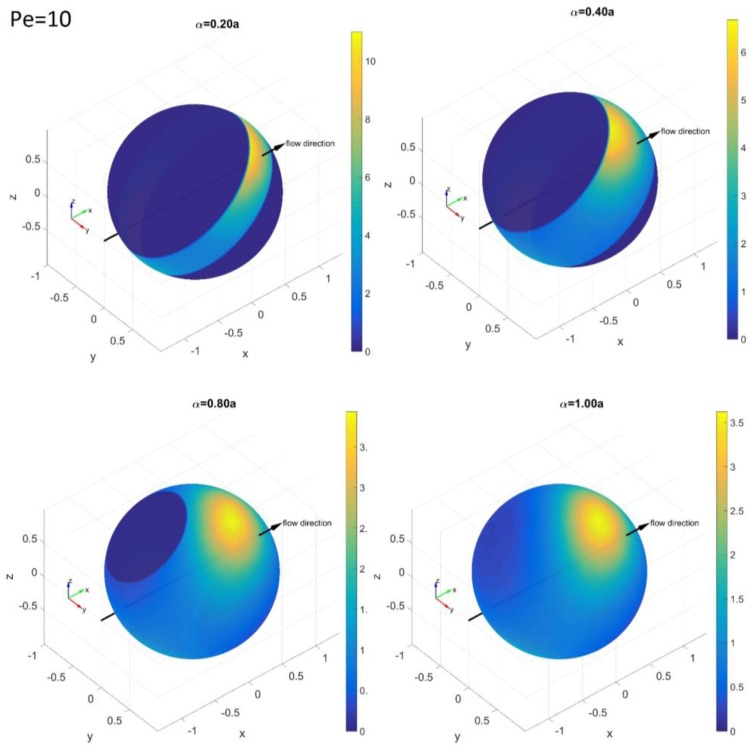
Simulation results for the PDF(*θ*,*ϕ*) on the spherical surface of the tips of a rod at *Pe* = 10 with *α*/*a* = 0.2, 0.4, 0.8 and 1.0. The color bars represent the probability density levels of each PDF from yellow (highest probability) to dark blue (lowest probability) (color online).

In contrast to the PDFs at low *Pe* values where Brownian rotation makes the distribution even within a confined angle region, PDFs at higher *Pe* values show distinctive concentrated densities on a certain angle region. We chose to present the results at *Pe* = 10 for the convenience of describing this distinctive feature. Figure 9 shows the spherical PDF(*θ*,*ϕ*) at *Pe* = 10. The unconfined PDF(*θ*,*ϕ*) at *α*/*a* ≥ 1 shows a concentrated density along the *x*-axis; however, it is shifted towards the *y*-axis. This distinctive distribution of Brownian rods under shear flow at *Pe* > 1 is explained by Jeffery orbit rotation, as well as the competition between rod orientation relaxation from the Brownian rotation and rod alignment from shear flow [21].

At *α*/*a* = 0.8, the confinement does not affect the maximum density region. Therefore, the PDF(*θ*,*ϕ*) is only sliced at the confinement and the overall shape is not changed much. However, as *α*/*a* becomes smaller than 0.4, the maximum density region at *α*/*a* > 0.4 begins to reside out of the confinement region. As a result, the distribution becomes more concentrated towards one side of the confinement region. 

**Figure 10:**
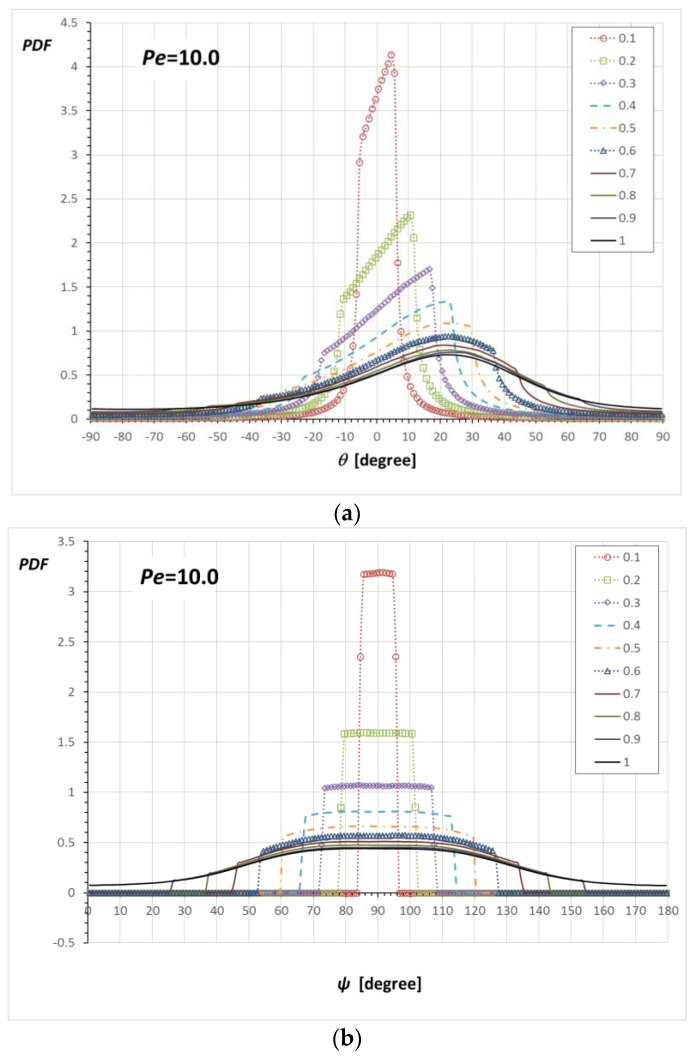
Simulation results for the (**a**) PDF(*θ*) and (**b**) PDF(*ψ*)/sin*ψ* at *Pe* = 10 with various *α*/*a*. It is seen that *θ*_max_ ≈ 25°, which corresponds to *α* = 0.43*a*.

Figure 10a shows the PDF(*θ*) at *Pe* = 10 and various *α*/*a*’s. At *α*/*a* = 1, where rod rotation is not restricted by a wall, the PDF(*θ*) shows the off-center maximum, which is well known for a Brownian rod under shear flow [21]. The off-center maximum is found to be at *θ*_max_ ≈ 25° for *Pe* = 10. As *α*/*a* is reduced and the confinement angle region remains larger than *θ*_max_ < sin^−1^(*α*/*a*) (i.e., 0.43 < *α*/*a* < 1), the off-center maximum is not affected but the distribution is sliced at sin^−1^(±*α*/*a*). However, at *α*/*a* < 0.43, the distribution becomes concentrated at the positive limit of the confinement, which is expected because the rod cannot be distributed towards the maximum density region at the unconfined condition. Figure 10b shows the PDF(*ψ*)/sin*ψ* at *Pe* = 10. The unconfined PDF(*ψ*)/sin*ψ* at *Pe* = 10 shows a curved distribution. As in the case of the PDF(*θ*), the PDF(*ψ*)/sin*ψ* at 0.43 < *α*/*a* < 1 shows the cutoff at sin^−1^(±*α*/*a*), whereas the PDF(*ψ*)/sin*ψ* at 0 < *α*/*a* < 0.43 shows square-like shape as in the low *Pe* case. 

Note here that PDFs at *Pe* = 1.0 simply show that the distribution patterns are in between those of *Pe* = 0.001 and *Pe* = 10.0. For example, the off-center maximum is found to be at *θ*_max_ ≈ 40.5° for *Pe* = 1.0. The confinement, sin 40.5° = 0.65 < *α*/*a* < 1, gives PDF(*θ*)s which maintain *θ*_max_ ≈ 40.5°, while the other confinement, *α*/*a* < 0.65, results in the distribution being concentrated at the positive limit (data not shown).

**Figure 11:**
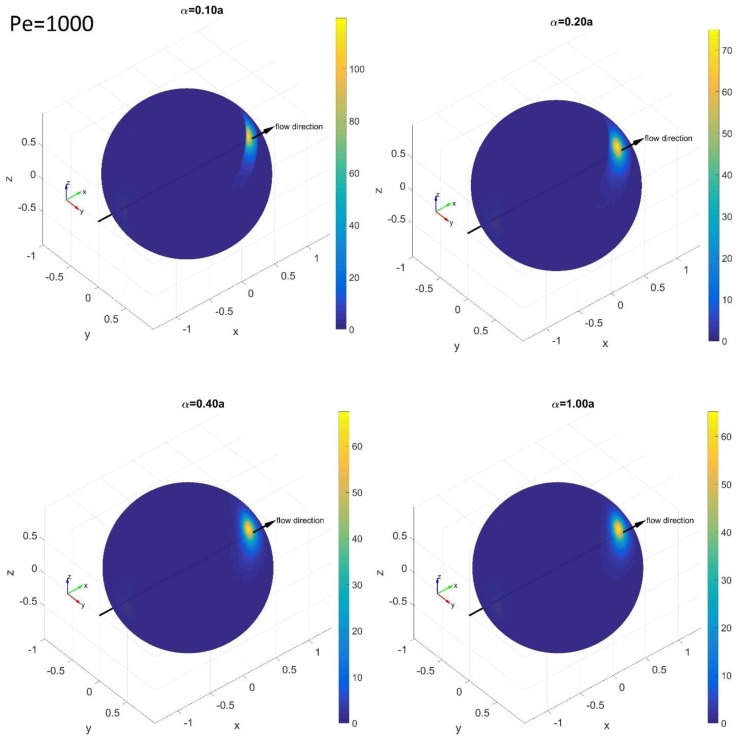
Simulation results for the PDF(*θ*,*ϕ*) on the spherical surface of the tips of a rod at *Pe* = 1000 with *α*/*a* = 0.1, 0.2, 0.4 and 1.0. The color bars represent the probability density levels of each PDF from yellow (highest probability) to dark blue (lowest probability) (color online).

**Figure 12:**
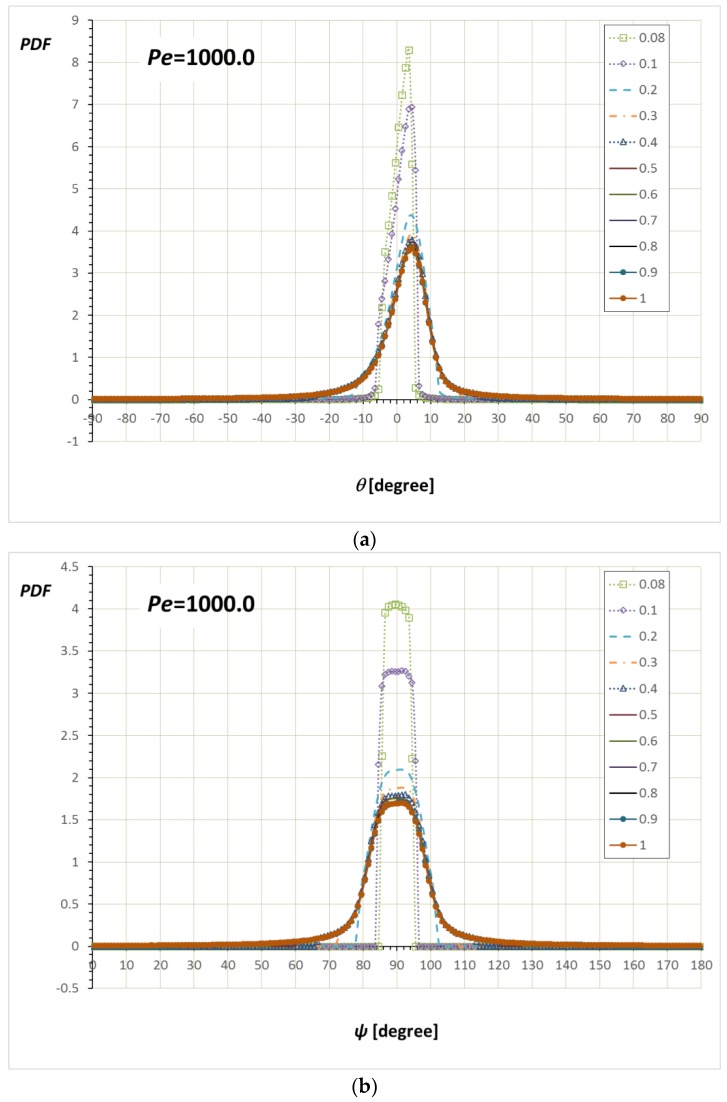
Simulation results for the (**a**) PDF(*θ*) and (**b**) PDF(*ψ*)/sin*ψ* at *Pe* = 1000 with various *α*/*a*. It is seen that *θ*_max_ ≈ 4.5°, which corresponds to *α* = 0.078*a*.

Figure 11 and Figure 12 show PDFs at *Pe* = 1000. At this high value of *Pe*, most of the distributions are aligned along the *x*-axis with the off-center maximum at *θ*_max_ ≈ 4.5°. The wide range of the confinement, sin 4.5° = 0.078 < *α*/*a* < 1, gives PDF(*θ*)s which maintain *θ*_max_ ≈ 4.5°. As in the PDFs shown so far, the pattern change happens when the confinement becomes narrower than the *θ*_max_ (sin 4.5° = 0.078 > *α*/*a*).

Comparing with the previous work by Hijazi and Khater [29], our PDF(*θ*) seems reasonably similar. Although the previous work used a different method for normalization and presented PDF(*θ*)s only at *Pe* = 2 and *Pe* = 200, qualitatively it is enough to compare our results inferred between *Pe* = 0.001 and *Pe* = 10 as well as between *Pe* = 10 and *Pe* = 1000. For the PDF(*θ*) at low *Pe*, the trend of the shape of the PDF(*θ*) being sliced at confinement appears the same. For the PDF(*θ*) at high *Pe*, the overall trends also seem the same, except *α*/*a* = 0.2. The difference is unclear due to the normalization method used in the previous work. Additionally, it should be pointed out that our PDFs are based on 3D simulation, whereas the previous work was based on 2D simulation. 

### 3.2. Average Orientation Moments near a Wall

**Figure 13:**
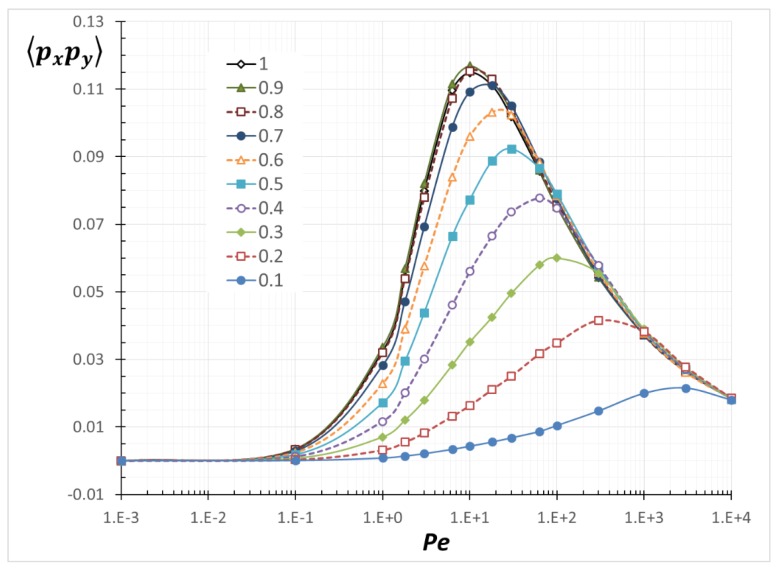
Average orientation moment <*p*_x_*p*_y_> as a function of *Pe* with various *α*/*a*.

**Figure 14:**
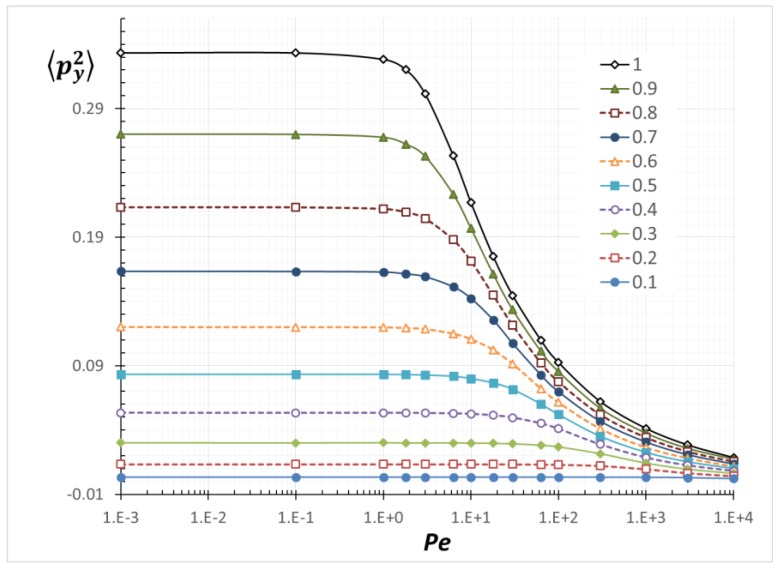
Average orientation moment <*p*_y_^2^> as a function of *Pe* with various *α*/*a*.

**Figure 15:**
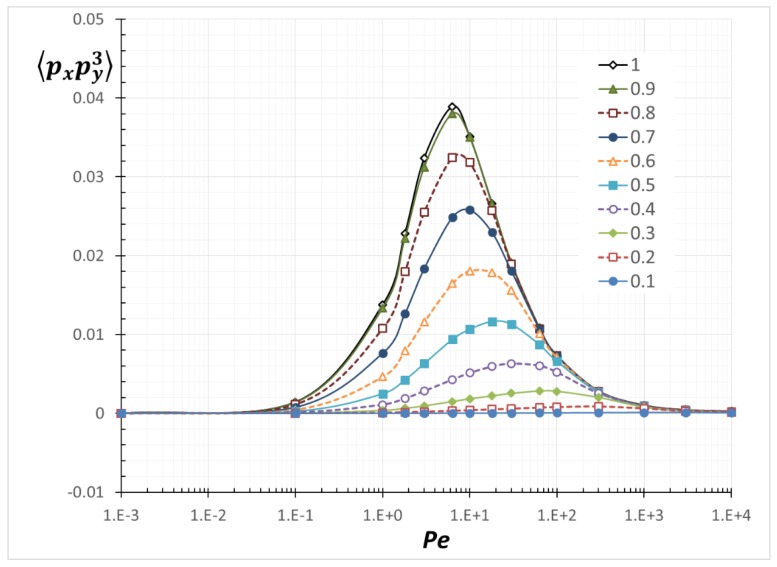
Average orientation moment <*p*_x_*p*_y_^3^> as a function of *Pe* with various *α*/*a*.

Figure 13, Figure 14 and Figure 15 are resulting from the simulation performed and show the average orientation moments, <*p*_x_*p*_y_>, <*p*_y_^2^> and <*p*_x_*p*_y_^3^>, as a function of *Pe* for various values for *α.* The average orientation moments at *α*/*a* = 1 (unbounded) reproduce previously determined results [26]. As *α* decreases (more confined), all the values are decreased. As can be inferred from Equation (3), <*p*_x_*p*_y_> is related to the PDF(*θ*) and <*p*_y_^2^> is related to the PDF(*ϕ*). As a PDF is narrowed by confinement, the related average orientation moments are reduced. The relations among *Pe*, *α* and each orientation moment in Figure 13, Figure 14 and Figure 15 can be used to calculate any transport variables of rods near boundaries. Although no formulas to express all of the values in the entire *Pe* and *α* ranges have been derived, interpolation between the obtained data points can give reasonable approximation to the values at arbitrary *Pe* and *α*. One application of utilizing the orientation moments is demonstrated in the next section.

### 3.3. Application to Improving a Shear-Induced Migration Theory

**Figure 16:**
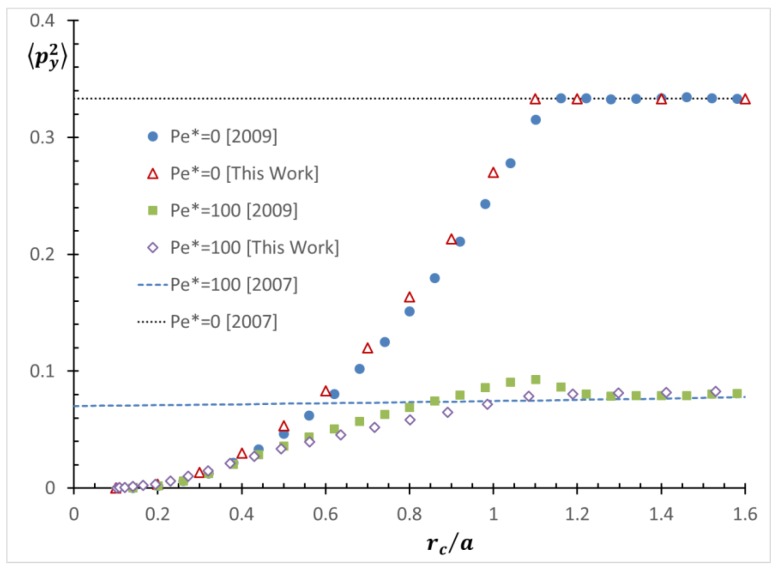
The <*p*_y_^2^> profile as a function of *r*_c_/*a* for a shear flow in a channel with *H* = 12*a*. Predictions from this work [open symbols], from the previous model [lines] by Park et al. (2007) [25] and the previous simulation results [solid symbols] by Park and Butler (2009) [24] are compared. Note that the half rod length distance from a wall is at 1.1*a* due to the rod diameter. The small discrepancy between the previous bulk values at *r*_c_ > 1.1*a* and the values from this work are from the interpolation.

A previous model equation for a shear-induced migration of a rod-like particle under shear flow near a boundary [25] did not consider the rod orientation dependence on the wall steric effect in the near-wall region. Therefore, the rod configurations in the near-wall region predicted by the model equation showed discrepancy from the result from the previous simulation. For example, Figure 16 compares the profiles of <*p*_y_^2^> as a function of *r*_c_/*a* for the case of Pe* = 0 (no flow), as well as a pressure-driven flow with Pe* = 100 in a microchannel of *H* = 12*a*. Note that this assigned value of Pe* for a pressure-driven flow is based on the cross-sectional average shear rate in the channel. Therefore, we distinguish the local *Pe*(*y*), which is dependent on *y*-position for pressure-driven flow:

(7) $$Pe\left(y\right)=2Pe^{*}\left|\frac{2y}{H}-1\right|$$

Since the previous model did not consider the wall confinement effect on the orientation distribution, the values of <*p*_y_^2^> in the channel were assumed to follow *Pe*(*y*) from Equation (7), even near the wall (see Figure 15). However, the previous simulation showed the reduction of <*p*_y_^2^> values at *r*_c_ < 1.1*a*, due to the wall confinement. The reason why the confinement region is *r*_c_ < 1.1*a* and not *r*_c_ < 1.0*a*, is due to the assumption that the closest position where the rod tip can be located in the previous simulation was set to *r*_c_ = 0.1*a*, considering its diameter. 

Our new prediction of <*p*_y_^2^> in Figure 16 can be applied to predict the <*p*_y_^2^> distributions in the channel. The results are also compared with the previous results in Figure 16. As mentioned in an earlier section, our prediction is shifted by the same amount in order to match the closest available position. The profiles of <*p*_y_^2^> obtained through our new results match those from the previous simulation for Pe* = 0. This indicates that our assumption made in our proposed simulation method is valid for the low *Pe* condition. 

The newly predicted profile of <*p*_y_^2^> at Pe* = 100 shows good agreement with the previous simulation results at *r*_c_ < 0.9*a*. We believe that this is the first time the orientation moments near a wall have been calculated. Furthermore, this result shows that our orientation moment which was calculated under simple shear flow can be applied to pressure-driven flow. This also supports Stover and Cohen’s argument [16], that shear gradient in pressure-driven flow does not affect the orientation distribution. However, there is some quantitative disagreement around *r*_c_ = 1.1*a*, as the values of <*p*_y_^2^> from the previous simulation are slightly higher. This discrepancy can be explained by the pole-vault motion. As shown in Figure 5, the *p*_y_ component becomes larger while the pole-vault motion results in an increasing *r*_c_, which results in the increase of <*p*_y_^2^> values. Since this effect is not considered in our simulation and the pole-vault motion only happens under shear flow, it can be inferred that the pole-vault motion was the cause of the bumps in the curvature of the graphed simulation results. Although some discrepancy was detected around *r*_c_ = 1.1*a* and high *Pe*, we claim that that discrepancy is not severe and our model can predict the rod orientation fairly well in the near-wall region.

## 4. Conclusions

We investigate the wall confinement effect on the orientation distribution for a rod near a wall (within a half rod length distance from a wall) under a shear flow. Brownian dynamics simulations were performed by only considering the rod rotation with given various values of *Pe* and *α*. This simulation method is proposed based on the findings from previous simulation studies that rod-wall hydrodynamic interaction did not affect the orientation distribution and the rod-wall collision causes the rod translation not the rod rotation.

The simulation results were analyzed to give the orientation angle distributions, Jeffery orbit distributions and the average orientation moments for various values of *Pe* and *α*. The PDF(*θ*) showed that if a wall confinement (sin^−1^(*α*/*a*)) is smaller than the characteristic *θ*_max_, then the distribution becomes concentrated at sin^−1^(*α*/*a*). The average orientation moments values were decreased with more confinement compared to the values under non-confinement (*α*/*a* ≥ 1.0).

The average orientation moments obtained from this study were applied to improve a shear-induced migration theory for rod-like particles in a microchannel flow. The original theory did not take into consideration the wall confinement effect on the orientation moments. Comparison of the orientation moment distribution in the cross-sectional direction from the new prediction and the previous simulation confirmed the following: (1) The rod translation due to Brownian collision does not affect the rod orientation, which agrees with the finding by Hijazi and Khater [28,29]. (2) The pole-vault motion slightly affects the rod orientation near the position of the half rod length but not to a severe level. Future calculations of the orientation moments in this study will be improved by considering the pole-vault motion, as well as details of rod shape, such as spheroid or cylinder. 

The orientation distribution and moments newly obtained from our study can be applied to improve the prediction of flow behaviors or structural configurations of rod-like particle in various flow systems. The model equations in the shear-induced rod migration theory and the subsequent theories on particle separation contains the terms of the average orientation moments [12,13]. A typical approach for evaluating the particle distribution in a flow system is to use the convective-diffusion equation, where diffusivity is usually assumed to be isotropic and constant in the channel [41].

## Abbreviations

**Notation**	
*a*	the half-length of the long principal axis of a rod
*b*	the half-length of the short principal axis of a rod
*d*	the length of the short principal axis (diameter or thickness) of a rod
*D**_R_*	Rotational diffusivity of a rod
***I***	identity matrix
*k**_B_*	Boltzmann constant
*L*	the length of the long principal axis of a rod
*m*	index of the sample time
*M*(*n*)	the total number of sampled orientation data for the *n*-th particle.
*n*	index of a particle
*N*	the number of particles in each set of simulation
*r_c_*	the rod center-of-mass position
**p**	rod orientation vector with a magnitude of unity
*p*_i_	the i-direction component of **p**
PDF	probability distribution function (normalized so that its integration gives 1)
*Pe*	rotational Peclet number
*Pe**	rotational Peclet number averaged over cross section for a pressure driven flow
*Pe*(*y*)	local rotational Peclet number at a cross sectional position *y* in a pressure driven flow
*t*	dimensionless time
*t*_m_,_n_	the *m*-th sampling time for the *n*-th particle
T	Brownian torque
*T*	Absolute temperature of the flow
*w*	random vector with zero mean and variance of 1
$\hat{x}$	a unit vector in the *x*-direction
*x*	flow direction in the Cartesian coordinate system
*y*	shear direction in the Cartesian coordinate system
*z*	vorticity direction in the Cartesian coordinate system
**Greek Letters**	
$\dot{\gamma }$	shear rate
*α*	wall confinement (distance from the wall surface to the rod center-of-mass position)
*θ*	the angle between a rod’s principal axis and the flow direction on the *xy*-plane
*ϕ*	the angle between a rod’s principal axis and the vorticity direction (*z*)
*χ*	the angle between a rod’s principal axis and the flow direction on the *xz*-plane
*ψ*	the angle between a rod’s principal axis and the shear direction (*y*)
*μ*	solvent viscosity
